# Determinants of severe acute malnutrition among children aged 6—23 months in bahir dar city public hospitals, Northwest Ethiopia, 2020: a case control study

**DOI:** 10.1186/s12887-022-03327-w

**Published:** 2022-05-20

**Authors:** Tigist Gebremaryam, Desalegne Amare, Tilksew Ayalew, Agimasie Tigabu, Tiruye Menshaw

**Affiliations:** 1 College of Medicine and Health Sciences, Debre Markos University, Debre Markos, Ethiopia; 2 School of Health Sciences, Bahir Dar University, Bahir Dar, Ethiopia; 3 College of Medicine and Health Sciences, Debre Tabor University, Debre Tabor, Ethiopia; 4 College of Medicine and Health Sciences, Debre Markos University, Debre Markos, Ethiopia

**Keywords:** Determinants, Under two children, Severe acute malnutrition, Ethiopia

## Abstract

**Background:**

Severe acute malnutrition is a major problem among developing countries and it is one of the major causes of mortality and morbidity in Ethiopia. The impact is more severe among children aged 6–23 months. Severely malnourished children are nine times more likely to die than healthy children. Identification of the determinants of severe acute malnutrition under the age of two years can significantly reduce the burden of child morbidity and mortality. Therefore, this study was aimed to assess determinants of severe acute malnutrition among children aged 6–23 months at Bahir Dar city public hospitals, Northwest Ethiopia, 2020.

**Methods:**

Institutional-based unmatched case–control study was conducted among a total sample size of 201 children (67 cases and 134 controls) in Felege Hiwot Comprehensive Specialized Hospital and Tibebe Ghion Specialized teaching hospital, from February 2020–March 2020. Children diagnosed with severe acute malnutrition were considered as cases and children with other problems were control groups. The study participants were randomly selected from pediatrics units in the two specialized hospitals. Data were collected using a structured pretested questionnaire through interviews and anthropometric measurements. The data were entered into Epi data version 3.1 and exported to SPSS software version 23 for analysis. Variables with (*p* < 0.25) in the bivariable analysis were entered into multivariable logistic regression. For multivariable analysis, a backward method was selected with a 95% confidence interval. Statistical significance was declared at *P* < 0.05.

**Results:**

In this study**,** 67 cases and 134 controls of children with their mothers had participated with an overall response rate of 100%. Family size > 5 [(AOR = 3.89, 95% CI:(1.19, -12.70)], average perceived birth weight [(AOR = 0.048, 95% CI: 0.015, -0.148)] and large perceived birth weight [(AOR = 0.023, 95% CI:(0.002, -0.271)], introduction of complementary feeding before six months [(AOR = 6.21, 95% CI: (1.44, -26.76)] and dietary diversity score < 5 groups [(AOR = 9.20, 95% CI; 3.40, -19.83)were significant factors associated with severe acute malnutrition.

**Conclusion:**

In this study, dietary diversity, family size, perceived birth weight, and initiation of complementary feeding were significantly associated with severe acute malnutrition. Therefore, emphasis should be given to improving infant and young child feeding practices, especially timely initiation of complementary feeding and dietary diversity.

**Supplementary Information:**

The online version contains supplementary material available at 10.1186/s12887-022-03327-w.

## Background

Malnutrition is broadly categorized as undernutrition and overnutrition. Undernutrition is a great concern in many developing countries including Ethiopia; it mainly occurs when an individual does not get enough food that consists of sufficient and balanced food groups. Undernutrition in children is classified as wasting (acute), stunting (chronic), or underweight [1].

Severe acute malnutrition (SAM) or severe wasting is defined by low Weight for Length (WFL) less than -3 standard deviation (SD) of the median World Health Organization (WHO) growth standards, and/or a low mid-upper-arm-circumference (MUAC) < 11.5 cm and/or presence of bilateral pitting edema [2]. SAM is the most extreme and visible form of acute undernutrition, resulting from acute food shortages, inadequate or poor quality of diet, a recent bout of illness, repeated infection, inappropriate child care or feeding practices, or a combination of these factors, which results in a child who loses weight rapidly and does not gain enough weight relative to his/her height [3].

Wasting in children is a life-threatening result of poor nutrient intake and/or disease. Children suffering from wasting have weakened immunity, are susceptible to long-term developmental delays, and face an increased risk of death, particularly in cases of severe wasting. These children require urgent treatment and care to survive. In 2019, 47.0 million children under 5 were wasted of which 14.3 million were severely wasted [4].

SAM can occur in all age groups, but the impact is more severe among children aged 6–23 months, as this period is extremely important for the child's optimal growth and development, due to this mental and physical damages that are difficult to reverse can occur due to nutritional deficiencies [5]. Brain and nervous system development begins early in pregnancy and is largely complete when the child reaches the age of two. The timing, severity, and duration of nutritional deficiencies during this period affect brain development, physical growth, and overall health of the child [6]. Globally, 83% of children less than two years are not getting enough nutritious food appropriate for their age, this denies the infant and young children the nutrients they need at the most critical time in their physical and mental development [7]. Infants and young children, especially, up to two years of age have the highest nutrient needs than any time in life. But many young children do not reach their full developmental potential; the main reason is that they are receiving too little food which may be inadequate, or too late /too early that is not provided timely [8].

The burden of diseases and death due to SAM in children is higher and more serious than any other cause. Breastfeeding with complementary feeding could reduce mortality among under-five children by 19% [9]. Complementary feeding is a time of transition from exclusive breastfeeding to family foods, typically covering the period from 6 to 23 months of age. It is times when malnutrition starts in many infants and young children and contributes to the high prevalence of malnutrition in children less than two years of age worldwide [10].

Worldwide, an estimated 16 million children under the age of 5 are affected by severe acute malnutrition with more risk to death than well-nourished children. These deaths are the direct result of malnutrition itself, as well as the indirect result of childhood illnesses like diarrhea and pneumonia that malnourished children are too weak to survive [11]. Globally, around 50 million children are acutely malnourished of which 17 million are severely wasted. Undernutrition is estimated to be associated with around half that is 45% of child deaths annually [12]. The first two years of a child’s life are especially important, as optimal nutrition during this period lowers morbidity and mortality, reduces the risk of chronic disease, and is the basis for better growth and development [13].

SAM in children is a major public health problem in developing nations, including sub-Saharan Africa [14]. Results from the 2019 Ethiopian mini Demography and Health Survey (EMDHS) show that in Ethiopia 37% of children under 5 are stunted, 12% are severely stunted, 7% are wasted, and 1% is severely wasted, 21% of all children are underweight, and 6% are severely underweight. There are also regional variations. Amhara regions are highly affected by child stunting 41.3% and 7.6% are wasted, including 1.7% who are severely wasted which is greater than the national level [15].

The National Nutrition Program of Ethiopia in 2016 was planned to reduce all forms of malnutrition; Stunting to 26.3%, Wasting to 4.9%, and Under-weight to 13.3%, and to increase the proportion of children 6–23 months received minimum dietary diversity to 56% in 2019/20. But, all plans are not achieved. So it is important to address determinants associated with SAM for better intervention [16].

The United Nations Children’s Emergency Fund (UNICEF), in its malnutrition framework, three major determinants that cause child undernutrition, is: basic or structural, underlying, and immediate determinants. Inadequate dietary intakes with diseases are considered the immediate causes. Poor socioeconomic status, household food insecurity, inadequate sanitation, and inaccessible health care are considered as underlying causes. Cultural barriers, political and socio-economic structures, and religious ideologies are basic-level causes (1).

Globally different literature revealed that socio-economic factors such as income, residence, occupation, education, maternal age, and family size [17] have influenced the occurrence of SAM. Community determinants such as lack of maternal and child health services, lack of adequate and safe water supply, and lack of improved environmental sanitation are other determinants [18, 19]. Child-related determinants such as a child’s gender, age, weight at birth, and dietary diversity [20] have been identified as individual-level determinants for SAM. Determinants associated with SAM may differ among Regions and Communities, as well as over time. The association of socio-demographic, maternal, and child health-related determinants that influence occurrences of SAM is not investigated in children under the age of two years in the study area. There are significant gaps in information on the association of SAM with complementary feeding practices in terms of: time of initiation, minimum dietary diversity and minimum meal frequency, child’s age, sex, perceived birth weight, mother’s BMI, mothers education, maternal marital status, family size, residence, source of drinking water, maternal ANC follow up, non-exclusive breastfeeding and child morbidity, vaccination status. Thus, this study was conducted to identify determinants of SAM among children aged 6–23 months at Bahir Dar city public hospitals, Northwest Ethiopia.

## Materials and methods

### Study area and period

The study was conducted in Bahir Dar city public hospitals; Felege Hiwot Comprehensive Specialized Hospital (FHCSH) and Tibebe Ghion Specialized teaching hospital (TGSTH), Amhara Region, Ethiopia, from February 2020 to March 2020. Bahir Dar city is the capital of the Amhara region, located approximately 565 km northwest of Addis Ababa (the capital city of Ethiopia). The city administration has 3 hospitals, 10 health centers, and 134 private health institutions. FHSCH is situated in Bahir Dar city kebele 13. TGSTH is located 10 km south of the city center. According to information obtained from administrative offices of these hospitals, they provide different services in the outpatient department, inpatient department, and operation room theater department. TGSTH and FHCSH serve more than 3.5 million and 5 million populations in their catchment area respectively. FHCSH has 14 physicians, 4 pediatricians. 33 nurses, and 61 beds in pediatrics unit with total annual under two admission of more than 988 of which more than 350 was due to SAM. TGSTH has 3 physicians, 2 pediatricians, 21 nurses, and 55 beds in pediatrics unit with total annual less than two admissions of more than 847 of which more than 300 were diagnosed with SAM.

### Study design and population

Institutional based unmatched case control study was conducted among cases (children 6–23 months of age with SAM) and controls (children 6–23 months of age without SAM) admitted in the pediatric ward of Bahir Dar city public hospitals of Northwest Ethiopia. All children 6–23 months of age with SAM or without SAM who were admitted to the pediatric ward were our study populations.

### Cases

Presence of one of the three clinical signs and criteria. Children with clinical signs of possible SAM according to WHO’S Integrated Management of Neonatal and Childhood Illness (IMNCI) guidelines, defined as children aged 6–23 months with severe acute malnutrition whose MUAC < 11.5 cm, or WLZ < -3 SD or with bilateral pitting edema (based on pediatrician’s assessment) and who were admitted to pediatrics unit of the two public hospitals of Bahir Dar city.

### Controls

Children age 6–23 months with normal nutritional status (without bilateral pitting edema or MUAC > 12.5 cm, or WLZ ≥ -2 SD), who did not fulfill SAM criteria and who were admitted with other health problems other than SAM in the two public hospitals of Bahir Dar city. The diagnosis includes history taking, clinical manifestations (objective findings) and anthropometric measurements.

### Sample size determination and sampling procedure

The sample size was determined using Epi-Info version 7. The double population proportion exposure difference formula was used by using major determinant variables (not a model by health extension program, not exclusively breastfed, not given colostrum, be bottle-fed and have illness during the last two weeks before the survey) from another study. Considering not exclusively breastfed (children introduced other diets before six months of age compared to those who did not) as independent predictor exposure variable since it gave the maximum sample size. From that study, the proportion of lack of exclusive breastfeeding is 43.7% among cases and 21% among controls [21]. Controls to case ratio of 2:1 were recruited to achieve 84% power at 5% significance level. Adding 10% non-response rate, the total sample size was 201 children with 67 cases and 134 controls. Cases were selected randomly among children in the age of 6–23 months admitted due to SAM in the therapeutic feeding units during the study period. Once a case child was admitted, his/her mother/caretaker was interviewed immediately and then the first two controls were selected by simple random method on the same day and in the same hospital.

### Data collection tools and quality assurance

The data collection tool was first developed in English and was translated to local language (Amharic) and was translated back to English to maintain consistency. The review was made by Amharic, English language experts and health professionals for consistency of language translation. A pretest was conducted on 5% of the total sample size out of the study area in Debre tabor general hospital, which is found in the south Gondar zone, before actual data collection and necessary adjustment was made on the tool. Two days training was given for data collectors and supervisors about anthropometric measurements and techniques of interview. A questionnaire was adapted from the World Health Organization instrument for stepwise surveillance for child malnutrition (WHO STEPS) [22] and by reviewing different literatures which were related to determinants of SAM.

Data were collected by four trained BSc nurses and two supervisors through face to face interview of the index mothers using pretested structured and interviewer administered questionnaires and anthropometric measurements [2] of the children(weight, length and MUAC).After measuring the weight and length of the child, we compute the WLZ index. (Mothers were interviewed about their socio-demographic characteristics, maternal determinants, environmental determinants and child related determinants in each pediatrics unit in the two public specialized hospitals. The outcome variable was SAM of the children.

## Measurements

Weight: weight measurements were taken to the nearest 0.1 kg using an electronic digital weight scale. The scale was checked before each weighing to ensure that the mark returned to zero. The children were weighted with light clothing and no shoes. The parent or caregiver stands on the scale first, without the child. Then measure the adult with the child and record immediately. Weights were taken in kilograms. Each child was weighed twice.

### Length

Measurement of supine or recumbent length was taken to the nearest 0.1 cm using a portable calibrated board. The sole of the baby's feet were held firmly against the wall at the zero point while the length was marked off on the chart at the crown of the head. Length of the child was measured in recumbent position without shoes.

To improve measurements accuracy we have used appropriate instruments and the data collectors were trained and know how to use these instruments properly and also by repeated measurement.

After anthropometric measurement of the children, WHO z-scores were computed.

To use the charts to classify children’s nutritional status: 1. Find the correct table for the child age (6–23 months) and sex (boy or girl). Measure children aged 6–23 months or less than 87 cm long lying down (length). 2. Find the figure closest to the child’s length in the left column. 3. Find the range that contains the child’s weight. 4. The label at the top of the column with the range containing the child’s weight tells the child’s nutritional status to classify as Cases (child with SAM) or controls (child without SAM).

To measure dietary diversity, we adopted the WHO and UNICEF Infant and Young Children Feeding guidelines (IYCF) as an internationally acceptable guideline [2]. Dietary diversity scores were estimated via a 24-h recall method by categorizing the food items into eight major food groups. The food groups assessed were; Grains, roots or tubers; Vitamin A-rich fruit and vegetables; other fruits and vegetables; Flesh foods (Meat, poultry, fish and seafood); Eggs; Legumes, Pulses or nuts, dairy products (milk and milk products) and breast milk. If a child consumed at least one food item from a food group throughout the previous day, the group was assigned a value of one (1) for that child, and zero (0) if not consumed. The group scores are then summed up to obtain dietary diversity score, which ranges from zero to eight, whereby zero represents the non-consumption of any of the food items in the food groups, and eight represents the highest level of diet diversification. The MDD (minimum dietary diversity) was attained if a child had consumed five or more food groups out of the eight food groups over the previous day.

To assure the quality of the data, a properly designed, pretested questionnaire was used. Training was given for the data collectors and supervisors for two days.

### Variable definitions

#### Cases

Children with severe acute malnutrition whose MUAC ≤ 11.5 cm, WLZ < -3 SD or with bilateral pitting edema

#### Controls

Children with normal nutritional status (without bilateral pitting edema or their MUAC > 12.5 cm, their WLZ ≥ -2 SD)

#### Complementary feeding

The process of starting to give additional foods and fluids to the child in addition to breast milk

#### Minimum dietary diversity

Children 6–23 months of age who consumed foods and beverages from at least five out of eight defined food groups during the previous day. A child with a dietary diversity score (DDS) of less than five is classified as having poor dietary diversity; if DDS greater than or equal to five, it is considered having good dietary diversity.

#### Perceived birth weight

A child whose body weight less than 1500 g at its birth is considered as Very small, A child whose body weight less than 2500 g at its birth considered as Small, A child whose body weight between 2500 g-4000 g at its birth considered as Average, A child whose body weight greater than 4000 g at its birth considered as Large.

#### Vaccinated

A child who completes all EPI scheduled vaccine considered as fully vaccinated, a child who doesn’t complete all EPI scheduled vaccine considered as not vaccinated.

#### Exclusive breastfeeding

Breastfeeding while giving no other food or liquid, not even water for infants up to the age of 6 months.

#### Severe acute malnutrition

A child whose weight for length is below -3 SD of the median WHO reference values

### Data processing and analysis

Data was checked for completeness and consistency and then it was cleaned, coded and entered using Epi data version 3.1 and it was exported to SPSS software version 23 for analysis. WLZ was calculated and compared using the WHO 2006 Growth Reference Standard. A child who’s WLZ less than − 3 Standard Deviation (SD) from the reference population was classified as severe wasting (2). Descriptive statistics were used to describe the study population in relation to relevant variables. The association between SAM and exposure variables was analyzed by using binary logistic regression analysis. The model fitness was tested by the Hosmer–Lemeshow goodness-of-fit tests. A chi-square and odds ratio (OR) with 95% CI were used to assess the relationship between factors associated with the occurrence of child SAM. Then variables that had association in the bivariable model (*p* < 0.25) were entered and analyzed by a multivariable logistic regression model to identify the independent effect of different determinants for the occurrence of SAM. Statistical significance was declared at *P* < 0.05.

## Results

### Socio-demographic characteristics of the respondents

A total of 201 Children (67 cases and 134 controls) admitted in the pediatric ward were included in the study with a response rate of 100%. According to this study, the mean age of the children was 13.4 (SD ± 5.3) months. The mean age of mothers was 28.5 (SD ± 6.4) years. Most of the participants were from rural areas (55.2% cases and 48.5% controls) and more than half 37(55.2%) of cases and 71(53%) of controls were males. Most of the study participants (80%) were orthodox Christian religion followers. Concerning marital status of mothers, 58(86.6%) of cases and 115(85.8%) controls were married (Table 1).

**Table 1:** Socio-demographic characteristics of the study participants, Bahir Dar City Public Hospitals, Northwest Ethiopia, 2020

Variables	Category	Cases	Controls
		Frequency (%)	Frequency (%)
Maternal age	15–24	22(32.8)	37(27.6)
	25–34	26(38.8)	72(53.7)
	35–49	19(28.4)	25(18.7)
Marital status	Currently married	48(71.6)	106(79.1)
	Currently unmarried	19(28.4)	28(20.9)
Maternal level of education	No formal education	28(53.8)	39(33.9)
	Primary	4(7.7)	9(7.8)
	Secondary and above	20(38.5)	67(58.3)
Paternal level of education	No formal education	37(55.2)	47(35.1)
	Primary	8(11.9)	21(15.7)
	Secondary and above	22(32.8)	66(49.3)
Paternal occupation	Government employee	14(20.9)	40(29.9)
	Merchant	10(14.9)	26(19.4)
	Farmer	23(34.3)	32(23.9)
	Others ^a^	20(29.9)	36(26.9)
Residence	Urban	30(44.8)	69(51.5)
	Rural	37(55.2)	65(48.5)
Family size	≤ 5	43(64.2)	119(88.8)
	> 5	24(35.8)	15(11.2)
Age of child	6–11	33(49.3)	53(39.6)
	12–23	34(50.7)	81(60.4)
Sex of child	Male	37(55.2)	71(53)
	Female	30(44.8)	63(47)

^a^ private Organization, Daily laborer

### Maternal related determinants of SAM

This study revealed that the proportion of women who gave their first birth less than twenty years was higher in cases 37(55.2%) than controls 29 (21.6%). Most of the mothers in 38(56.7%) of cases and 121(90.3%) of controls had ever got ANC service during their pregnancy of the current child. The proportion of women who had attended ANC service less than three times is higher in cases 30(78.9%) than controls 50(41.3%). Regarding place of delivery more than half of women in cases 40(59.7%) and more than ninety percent of controls 126(94%) had given birth at health institution (Table 2).

**Table 2:** Distribution of maternal related determinants among the study participants, Bahir Dar City Public Hospitals, Northwest Ethiopia, 2020

Exposure variables	Responses	Cases	Controls
		Frequency (%)	Frequency (%)
Maternal age at first birth	15–19 20–49	37(55.2) 30(44.8)	29(21.6) 105(78.4)
Maternal ANC follow up	Yes No	38(56.7) 29(43.3)	121(90.3) 13(9.7)
Number of ANC services	≤ 3 > 3	30(78.9) 8(21.1)	50(41.3) 71(58.7)
Place of birth	Home Health institution	27(40.3) 40(59.7)	8(6) 126(94)

### Environmental related determinants of SAM

In this study, the families who had access to protected water sources for drinking 46(68.7%) were cases and 106(79.1%) were controls. Concerning cooking fuel, 15(22.4%) cases and 63(47%) controls used modern fuel. Households who have toilets were 34(50.7%) in cases and 109(81.3) in controls. Regarding methods of hand washing 32(47.76%) cases and 50(37.31%) controls were to wash their hands with water only (Table 3).

**Table 3:** Distribution of environmental related determinant among the study participants, Bahir Dar City Public Hospitals, Northwest Ethiopia, 2020

Exposure variables	Responses	Cases	Controls
		Frequency (%)	Frequency (%)
Source of drinking water	Protected Unprotected	46(68.7) 21(31.3)	106(79.1) 28(20.9)
Type of cooking fuel	Modern Traditional	15(22.4) 52(77.6)	63(47) 71(53)
Method of waste disposal	Proper Improper	23(34.3) 44(65.7)	94(70.1) 40(29.9)
Having toilet	Yes No	34(50.7) 33(49.3)	109(81.3) 25(18.7)

### Child related determinants of SAM

In this study, the proportion of children with small perceived birth weight was higher in cases 48(71.6%) than controls 9(6.7%). Similarly the proportion of children who were not vaccinated at all was higher in cases 40(59.7%) than controls 27(20.1%). More than half of cases 47(70.1%) and less than one fourth of control 23(17.2%) not have been exclusively breast fed. Regarding the initiation of complementary feeding 44(65.7%) of cases and 19(14.2%) of controls started complementary feeding before six months. The proportion of children with dietary diversity scored less than five food groups was higher in cases 58(86.5%) than controls 29(21.6) based on 24 h dietary recall. Regarding morbidity factors of children 23(50.0%) cases and 2(11.1%) controls had a history of diarrhea and also 10(21.7%) cases and 9(50.0%) controls had a history of fever in the last two weeks (Table 4).

**Table 4:** Distribution of child related determinants among the study participants, Bahir Dar City Public Hospitals, Northwest Ethiopia, 2020

Exposure variables	Responses	Cases	Controls
		Frequency (%)	Frequency (%)
Perceived birth weight of the child at birth	Large Average Small	1(1.5) 18(26.9) 48(71.6)	19(14.2) 106(79.1) 9(6.7)
Getting nutritional information	Yes No	44(65.7) 23(34.3)	124(92.5) 10(7.5)
Did the baby sick in the last two weeks	Yes No	53(79.1) 14(20.9)	18(13.4) 116(86.6)
Child Vaccination status	Fully vaccinated Age appropriate Not vaccinated at all	23(34.3) 4(6.0) 40(59.7)	93(69.4) 14(10.4) 27(20.1)
Exclusive breastfeeding for 6 months	Yes No	20(29.9) 47(70.1)	111(82.8) 23(17.2)
Baby breast feed currently	Yes No	45(67.2) 22(32.8)	101(75.4) 33(24.6)
Time for introducing complementary feeding	At 6 month Before 6 month After 6 month	7(10.4) 44(65.7) 16(23.9)	43(32.1) 19(14.2) 72(53.7)
Dietary diversity score	< 5 food groups ≥ 5 food groups	58(86.5) 9(13.5)	29(21.6) 105(78.4)

### Determinants of severe acute malnutrition (SAM)

The multivariable logistic regression result showed that, large family size [(AOR = 3.89, 95% CI:(1.19, -12.70)], larger perceived weight at birth [(AOR = 0.023, 95% CI: (0.002, -0.271))], poor dietary diversity, [(AOR = 9.20, 95% CI; 3.40, -19.83) and initiation of complementary feeding before six month [(AOR = 6.21, 95% CI;(1.44,-26.76))] were significantly associated with SAM.

Family size was found to be significantly associated with SAM. Children from households with large family size > 5 were 3.89 times more likely to be affected by SAM [(AOR = 3.89, 95% CI; (1.19, -12.70))] as compared to children from households with smaller family size (≤ 5).

Perceived birth weight was found to be significantly associated with SAM. The odds of having SAM was 95% lower if the child had average perceived weight at birth [(AOR = 0.048, 95% CI; 0.015, -0.148)]. Similarly, the odds of having SAM was 98% lower if the child had a large perceived weight at birth [(AOR = 0.023, 95% CI;(0.002, -0.271)].

Dietary diversity was found to be significantly associated with the risk of SAM. Children who had poor dietary diversity (< 5 food groups) [(AOR = 9.20, 95% CI; (3.40, -19.83))] were 9.20 times more likely to be acutely malnourished as compared to children with good dietary diversity (≥ 5 food groups).

Time for the introduction of complementary feeding was significantly associated with the risk of SAM. The introduction of complementary feeding before six month [(AOR = 6.21, 95% CI: (1.44, -26.76))] increases the risk of SAM by 6.21 times as compared to complementary feeding started at six months (Table 5).

**Table 5:** Determinants of SAM among children aged 6—23 months in Bahir Dar city public hospitals, Northwest Ethiopia, 2020

Variables	Category	Cases	Controls	COR, 95% CI	AOR, 95% CI
		Frequency (%)	Frequency (%)		
Maternal level of education	No formal education	37(55.2)	47(35.1)	2.36(1.24–4.51)	
	Primary	8(11.9)	21(15.7)	1.14(0.44–2.95)	
	Secondary and above	22(32.8)	66(49.3)	1	
Family size	≤ 5	43(64.2)	119(88.8)	1	
	> 5	24(35.8)	15(11.2)	4.43(2.13–9.22)	3.89 (1.19–12.70)*
Type of cooking fuel	Modern	15(22.4)	63(47)	1	
	Traditional	52(77.6)	71(53)	3.08(1.58–5.99)	
Method of waste disposal	Proper	23(34.3)	94(70.1)	1	
	Improper	44(65.7)	40(29.9)	4.49(2.41–8.40)	
Having toilet	Yes	34(50.7)	109(81.3)	1	
	No	33(49.3)	25(18.7)	4.23(2.22–8.08)	
PBwt of the child at birth	Small	48(71.6)	9(6.7)	1	
	Average	18(26.9)	106(79.1)	0.03(0.013–0.076)	0.048(0.015–0.148)**
	Large	1(1.5)	19(14.2)	0.01(0.001–0.083)	0.023(0.002–0.271)*
Child Vaccination status	Fully vaccinated	23(34.3)	93(69.4)	1	
	Age appropriate	4(6)	14(10.4)	1.16(0.35–3.84)	
	Not vaccinated at all	40(59.7)	27(20.1)	5.99(3.07–11.68)	
EBF for 6 months	Yes	20(29.9)	111(82.8)	1	
	No	47(70.1)	23(17.2)	11.96(5.97–23.96)	
DD score	< 5 food groups	58(86.5)	29(21.6)	18.99(8.67–22.47)	9.20( 3.40- 19.83)**
	≥ 5 food groups	9(13.5)	105( 78.4)	1	
Time for introducing CF	At 6 month	7(10.4)	43(32.1)	1	
	Before 6 month	44(65.7)	19(14.2)	14.23(5.43–37.29)	6.21 (1.44–26.76)*
	After 6 month	16(23.9)	72(53.7)	1.37(0.52–3.58)	

Key: *PBwt* perceived birth weight, *EBF* exclusive breast feeding, *DD* Dietary diversity,

*CF* complementary feeding, ** = *p*-value < 0.001, * = *p*-value < 0.05

## Discussion

This case control study assessed determinants of SAM among 6–23 months children at Bahir Dar city public hospitals. In this study, the determinants identified for SAM were family size, perceived birth weight of the child at birth, introduction of complementary feeding and dietary diversity score.

Family size was found to be a significant predictor of SAM. Those children from households with large family size were found to be at significant risk of being acutely malnourished as compared to children from households with smaller family size. This finding is comparable with studies conducted in south omo [23], Karat [24]; which revealed that children who live in family size greater than five were more likely to develop acute malnutrition as compared with family size of less than three This is also in line with a study conducted in Ahmedabad India, children who live in family size greater than or equal to six were more likely to develop acute malnutrition than their counterpart [25]. This is due to the fact that as the family member increases the quality and time of care given to the child decreases which leads to SAM. Since large family members increase the burden of the scarce household resources to provide nutritious food to all family members and there is more competition for available food [26]. But, this result is contradicting to a study conducted in Patna India, which revealed that large household size is a protective factors against malnutrition in children [27].This is due to socio-economic and cultural difference, in our setup when families are large and their resources are limited, the available food is shared by all members, reducing the amount individuals get and care given to the children decreased [26].

Perceived birth weight of average and large were protective factors of SAM. Accordingly, children with average perceived weight at birth were 95% and children with large perceived weight at birth were 98% less likely to be malnourished as compared to children perceived as small at birth. This is consistent with a study conducted in Myanmar further analysis of 2015–16 DHS, which revealed the risks of undernutrition were higher among children perceived to have low birth size compared with children of average and above perceived birth size [19]. Another study in Malaysia also showed that households with low birth weight were at higher odds of having malnourished children as compared to average and above birth weight [28]. This might be due to maternal malnutrition during pregnancy which results in birth weight less than the average and child with malnutrition. In addition children born of well-nourished mothers are less likely to be wasted due to mother’s adequate intake of nutrients such as protein, energy, vitamin and minerals during pregnancy; such nutrients are important for the fetus to obtain average and large weight at birth [29].In other word nutritional status before and during pregnancy influences maternal and child outcomes. Optimal child development requires adequate nutrient intake, provision of supplements and prevention of disease. Maternal malnutrition leads to poor fetal growth and low birth weight [30].

Dietary diversity was found to be a significant factor of SAM. In this study children who had poor dietary diversity scores (< 5 food groups) were more likely to be acutely malnourished as compared with children with good dietary diversity scores (≥ 5 food groups). Similar findings were also documented in other studies done in Dabat [31] and Ghana [32]. This is due to the fact that poor dietary diversity is an indicator of poor quality of diet and nutrient intake of children and it negatively affects the nutritional status of children. During early life, the growth and development of the body are dependent on adequate supply of all essential nutrients. Providing nutrient-rich foods in sufficient quantity and quality starting from six months of age is one strategy to reduce child malnutrition. Providing good dietary diversity is also important to develop the immune system and prevent infections. So that poor dietary diversity may expose the child to infection due to low immunity, which may lead to severe acute malnutrition [24].

Time for the introduction of complementary feeding was found to be an important determinant of SAM. In this study the introduction of complementary feeding before six month increases the risk of acute malnutrition as compared to complementary feeding started at six months. This finding is also supported by other studies which are done in India [25], and Nepal [33]; initiation of complementary feeding before or after 6 months was found to be at risk of SAM. This is due to the early introduction of complementary food and is associated with an increased risk of gastro-intestinal and other infections. When complementary foods are started before six months, there is a reduction in breast milk consumption, which can lead to a reduction of immunity. When there is low immunity it can lead to infection and finally it results in SAM [26].

This study has the following strengths. Data were collected through face to face interviews which could be able to reduce information bias. To minimize recall bias the recalling period was made shorter for some variables. Efforts were made to choose the controls as randomly as possible. The study has the following limitations; since the questions relied on the memory of the mothers/ caretakers, this might introduce recall bias. There might also be selection bias because controls were selected from health facilities. Matching has a potential benefit in preventing confounding so this study could have limitations on addressing it. SAM tends to be seasonal, but we didn’t account for seasonal variation, this study has limitations on addressing seasonal variation.

## Conclusion

This study shows that, among children admitted in pediatrics units of the two hospitals, children who have family size of greater than five, birth weight perceived to be small at birth, complementary feeding started before six months, and dietary diversity score less than five were independent determinants of SAM among children 6–23 months. Therefore, Professionals working in child health service should provide simple and easy to understand information to the mother/caretaker on child caring practice and nutritional information including timely initiation of complementary feeding and appropriate diet diversity. Make family planning methods and information available for mother’s to manage family size. Interventions should be given during ANC follow up to improve maternal nutrient intake include supplementation with iron, folic acid or multiple micronutrients and provision of food and other supplements where necessary to prevent a child’s low birth weight. Future researches on child SAM are recommended to conduct community based longitudinal study integrating with qualitative study design on prospective dietary assessment.

## Supplementary Information

**Additional file 1:** 

## Data Availability

The datasets used and/or analyzed during the current study available from the corresponding author on reasonable request.

## Abbreviations

AOR: Adjusted Odds Ratio
CI: Confidence Interval
COR: Crude Odds Ratio
DDS: Dietary Diversity Score
EBF: Exclusive Breast Feeding
FHCSH: Felege Hiwot Comprehensive Specialized Hospital
IYCF: Infant and Young Child Feeding
MDD: Minimum Dietary Diversity
MUAC: Mid Upper Arm Circumference
SAM: Severe Acute Malnutrition
SD: Standard Deviation
SPSS: Statistical Package for Social Science
STEPS: Stepwise Surveillance for child malnutrition
TGSTH: Tibebe Ghion Specialized teaching hospital
UNICEF: United Nations International Children’s Emergency Fund
WHO: World Health Organization
WLZ: Weight for Length Z-score
