# Supplementary material for: Determinants of severe acute malnutrition among children aged 6—23 months in bahir dar city public hospitals, Northwest Ethiopia, 2020: a case control study
Source: BMC Pediatr. 2022 May 20;22:296. doi: 10.1186/s12887-022-03327-w (PMC9123747; doi:10.1186/s12887-022-03327-w)
Supplement: Supplementary file 1 — Additional file 1: [file 12887_2022_3327_MOESM1_ESM.docx]

# APPENDIX

**Appendix I: Information sheet**

Good morning/ afternoon?

My name is Tigist G/Maryam, I am graduate student at Bahir Dar University College Medicine and Health Sciences, Department of pediatrics and child health Nursing. And now I am conducting a study to assess determinants of severe acute malnutrition among children aged 6-23 months in Bahir dar city public hospitals, Northwest Ethiopia, 2020

**Title of the research**: Determinants of severe acute malnutrition among children aged 6-23 months in Bahir dar city public hospitals, North West Ethiopia, 2020

**Objective:** This study is aimed to identify determinants of severe acute malnutrition among children aged 6-23 months

**Participants**: Children aged 6-23 months admitted to pediatrics unit with their mother/caretaker in Bahir dar city public hospitals

**Potential Risks**: There is no foreseen risk by being involved in this study.

**Benefits:** No financial benefits are related with this study. But by participating in this study, most importantly, the result of the study will be beneficial to design effective preventive and control measures for severe acute malnutrition. Hence, you are indirectly benefiting other patients and the society in this respect.

I would like to ask you few questions. Your honest response to the questions can make the study to achieve its objective. All the information that you give will be kept confidential and private. Only the principal investigator and interviewer will have access to the information. You are kindly requested to respond voluntarily. You can also choose not to participate in this study totally or if you become uncomfortable during the interview, you will be allowed to leave at any time. At any time that you have questions, you can contact me by using the following Addresses**:**

Principal investigator: Tigist G/Maryam

Mobile phone: 0924268177

Email: [tigistgmariam12@gmail.com](mailto:tigistgmariam12@gmail.com)

**Appendix II- Consent Form**

In signing this document, I am giving my consent to participate in the study entitled “determinants of severe acute malnutrition among children aged 6-23 months in Bahir dar city public hospitals.”

I have been informed that the purpose of this study is to identify determinants of severe acute malnutrition. Thus, I have understood that participation in this study is entirely voluntarily. I have been told that my answers to the questions will not be given to anyone else and no reports of this study ever identify me in any way. I have also been informed that my participation or non-participation or my refusal to answer questions will have no effect on me. I understood that participation in this study does not involve risks. So are you willing to participate in this study?

1. If yes, proceed to the next page
2. If no, thank you, and skip to the next participant

Respondent’s signature_______________ Date of interview: _______________

Interviewer Name_______________________ Signature _________________

Supervisor’s name ________________ signature _____________

Results of interview questionnaire

1. Completed

2. Refused

3. Partially completed

**Appendix III- English version Questionnaire**

Bahir Dar University, college of medicine and health science, department of pediatrics and child health nursing

Questionnaire for the study of the determinants of severe acute malnutrition among children aged 6-23 months in Bahir dar city public hospitals

1. Questionnaire code number___________ 2. Name of health facility _____________
2. Status 1. **Case** 2. **Control**

Note: Encircle from the given options and write if any other idea or answer is given

| **PART I. Socio-demographic and economic characteristics of the family and child (6-23 months)** | | | | | |
| --- | --- | --- | --- | --- | --- |
| No. | | Question | Response | Skip | |
| 101 | | Mother’s age | ____________ (in years) |  | |
| 102 | | Marital status of mother | 1. Single 2. Married 3. Widowed 4. Divorced 5. Separated |  | |
| 103 | | What is your religion? | 1. Orthodox 2. Muslim 3. Catholic 4. Protestant 5. Other (specify) _________ |  | |
| 104 | | Residence | 1. Urban 2. Rural |  | |
| 105 | | Maternal level of education | 1. Can’t read and write 2. Can read and write 3. Primary school 4. Secondary school 5. College and higher |  | |
| 106 | | Paternal level of education | 1. Can’t read and write 2. Can read and write 3. Primary school 4. Secondary school 5. College and higher |  | |
| 107 | | Occupation of mother | 1. Housewife  2. Government employee  3. Merchant  4. Private Organization  5. Daily laborer  6. Other (specify) ____________ |  | |
| 108 | | Occupation of father | 1.Government employee  2.Merchant  3.Private Organization  4.Daily laborer  5. Other (specify) ____________ |  | |
| 109 | | Monthly income of the house hold | ____________ Ethiopian Birr |  | |
| 110 | | Family size | ________ (in number) |  | |
| 111 | | Age of the child | ________months |  | |
| 112 | | Sex of the child | 1. Male  2. Female |  | |
| **PART II. Maternal related determinants** | | | | | |
| 201 | Maternal age at first birth | | ____________( in year) |  | |
| 202 | Did you visit health facility for ANC during your pregnancy for this child? | | 1. Yes 2. No | If ‘no’ skip to ‘204’ | |
| 203 | If yes, for question ‘202’ How many times you visited health facility for ANC during the pregnancy? | | ____________ times |  | |
| 204 | Place of delivery | | 1.Home  2.Health institution  3. Other (specify) ____________ |  | |
| **PART III. Environmental related determinants** | | | | | |
| 301 | Source of drinking water | | 1. Spring 2. well 3. Tap 4. Other (specify)__________ |  | |
| 302 | If your source is not tap do you treating your water before drink in your home? | | 1. Yes 2. No | If ‘no’ skip to ‘304’ | |
| 303 | By what method do you wash your hand before preparing food and delivering food? | | 1.Always wash with water and soap  2. Always wash with water only  3. wash sometimes with soap |  | |
| 304 | Type of cooking fuel? | | 1. Firewood 2. Gas 3. Electric |  | |
| 305 | Method of waste disposal | | 1. Burning 2. Bury to pit 3. Open field |  | |
| 306 | What type of toilet do you have? | | 1. Temporary 2. Hygienic 3. No 4. Other(specify) ________ |  | |
| **PART IV-Child health and caring practice related determinants** | | | | | |
| 401 | Perceived birth weight /size of the child at birth | | 1. Very small 2. Small 3. Average 4. Large | |  |
| 402 | Did the child get Vaccination? | | 1. Fully vaccinated 2. Not-fully vaccinated 3. Not vaccinated at all | |  |
| Child morbidity status | | | | | |
| 403 | Did the baby have been sick within the last 2 weeks | | 1. Yes 2. No | If ‘no’ skip to ‘407’ | |
| 404 | If Yes for the above question ‘405’ Which? | | 1. Diarrhea 2. Malaria 3. Fever 4. Cough 5. Others(specify) ____________ |  | |
| Child feeding practice | | | | | |
| 405 | Do you get nutritional information on child feeding? | | 1. Yes 2. No | If ‘no’ skip to ‘409’ | |
| 406 | If yes for the above question ‘408’ from where do you get information? | | 1.From health institution  2.From media(radio/TV)  3.From newspaper or magazine |  | |
| 407 | Did your child breast feed exclusively for six months? | | 1. Yes 2. No |  | |
| 408 | Is the baby on breast feeding now? | | 1. Yes  2. No |  | |
| 409 | Time for introducing complementary feeding | | 1. Before 6 months 2. After 6 months 3. At 6 months |  | |
| 410 | Usual number of meals per day/  Minimum Feeding frequency | | 1. 2-3 times 2. 4-5 times 3. Above 5 times |  | |
| 411 | Food groups in 24 hours/  Dietary diversity score | | 1. < 5 food groups 2. > 5 food groups |  | |

|  |
| --- |

**24 hours dietary recall for the child**

What did your child eat yesterday (from morning when she/he woke up until bed time?)

| 1 | Bread, rice, biscuit, roasted-grain, maize, barely, wheat, teff, cereal or foods made from pulse | 1. Yes 2. No |
| --- | --- | --- |
| 2 | Potato, sweet potato, foods made from root or tuber | 1. Yes 2. No |
| 3 | Vegetables or foods made from vegetables | 1. Yes 2. No |
| 4 | Fruits or foods made from fruits | 1. Yes 2. No |
| 5 | Meat products; beef, lamb, goat, chicken | 1. Yes 2. No |
| 6 | Egg or foods made from egg | 1. Yes 2. No |
| 7 | Fish or foods made from fish | 1. Yes 2. No |
| 8 | Foods made from bean, pea, lentil, nut | 1. Yes 2. No |
| 9 | Milk, cheese, yoghurt and foods made from milk products | 1. Yes 2. No |
| 10 | Foods made with oil, fat, or butter? | 1. Yes 2. No |
| 11 | Foods made with honey, sugar | 1. Yes 2. No |
| 12 | Drinks with spice; tea, coffee | 1. Yes 2. No |
| 13 | Breast milk | 1.Yes 2.No |

| **Dietary Diversity Score for the child below two years** | | |
| --- | --- | --- |
| No. | From the foods reported in table…above, place 1 if there is any food consumed by the child and zero if the foods in that particular group were not consumed. | **Child (put1 if consumed, 0 if not consumed)** |
| 1 | **Grains, roots or tubers-** Any food from grain/cereals like rice, wheat, barley, oats, cornmeal(e.g. bread, pasta, breakfast cereals, grits , porridge)  Roots (carrots, onions, turnip, beets, beetroot) Tubers( yams, potatoes) |  |
| 2 | **Vitamin A-rich foods(**liver**,** salmon, butter, boiled egg, sweet potato, carrot, peas, red pepper, broccoli, tomato, spinach, lettuce, mango, grapefruit, watermelon, papaya, tangerine**)** |  |
| 3 | **Other fruits and vegetables-** Fruits (apples, oranges, grapefruits, bananas, mangoes, strawberries, avocados) Vegetables(lettuce, cabbage, potato, sweet potato, onion, garlic, asparagus) |  |
| 4 | **Flesh foods (Meat, poultry, fish and seafood) -** Any beef, lamb, goat, chicken, liver, kidney, heart, or other organ Meats? |  |
| 5 | **Eggs** |  |
| 6 | **Legumes, Pulses or nuts-** Any foods made from beans, peas, lentils, or nuts ,peanut, soybean, lupines, chickpeas, |  |
| 7 | **Milk and milk products-**Any cheese, yogurt, milk or other milk products? |  |
| 8 | **Breast milk** |  |
|  | Total |  |

**PART V: Anthropometrical measurement of the child**

| 1 | Child’s weight | ________Kilogram |
| --- | --- | --- |
| 2 | Child’s recumbent length | ________Centimeter |
| 3 | Child’s Mid upper arm circumference | ________Centimeter |

Z- Score

**Thank you!!**
